# Validation of the Patient Health Questionnaire (PHQ-9) as a screening tool for depression in pregnant women: Afaan Oromo version

**DOI:** 10.1371/journal.pone.0191782

**Published:** 2018-02-06

**Authors:** Yitbarek Kidane Woldetensay, Tefera Belachew, Markos Tesfaye, Kathryn Spielman, Hans Konrad Biesalski, Eva Johanna Kantelhardt, Veronika Scherbaum

**Affiliations:** 1 Institute of Biological Chemistry and Nutrition (140a), University of Hohenheim, Stuttgart, Germany; 2 Food Security Center, University of Hohenheim, Stuttgart, Germany; 3 Department of Population and Family Health, College of Health Sciences, Jimma University, Jimma, Ethiopia; 4 Tufts University, Freidman School of Nutrition Science and policy, Boston, United States of America; 5 Department of Gynecology, Faculty of Medicine, Martin-Luther University, Halle, Germany; 6 Institute of Medical Epidemiology, Biostatistics, and Informatics, Faculty of Medicine, Martin-Luther University, Halle, Germany; Auburn University at Montgomery, UNITED STATES

## Abstract

**Background:**

Semantic, technical, content, criterion and conceptual equivalence must be examined in order to validate a psychological rating scale in a new cultural setting. Few validation studies have been conducted in sub-Saharan Africa for scales seeking to detect depression in pregnant women. The aim of this study is to validate the 9-item Patient Health Questionnaire (PHQ-9) as a screening instrument for depression among Afaan Oromo speaking pregnant Ethiopian women.

**Methods:**

A random sample of 246 pregnant women were recruited in Seka Chekorsa District, Ethiopia during their first, second or third trimester. One week later, 29 participants were selected to answer the questionnaire for a second time to evaluate test retest reliability. The Mini International Neuropsychiatric Interview (MINI-Plus) scale was used as a gold standard to evaluate validity. PHQ-9 was compared with MINI-Plus and sensitivity, specificity, accuracy, positive likelihood ratio, negative likelihood ratio and Receiver Operating Characteristic Curves (ROC) for PHQ-9 were calculated. Rasch analysis was also carried out using Winsteps version 3.81.0.

**Results:**

The reliability coefficient, Cronbach's alpha, for the PHQ-9 total score was 0.84. Both the agreement and consistency Intra-class Correlation coefficients (ICC) for the one-week test-retest reliability were 0.98. The cut-off point of a summed score of eight resulted in a sensitivity of 80.8% and a specificity of 79.5%. The calculated area under the curve (AUC) for the PHQ-9 score versus the MINI-Plus was excellent, 0.88 (SE = 0.04; CI = 0.81–0.95). The PHQ-9 meets the criteria established by Linacre for rating scale effectiveness.

**Conclusions:**

The PHQ-9 proved to be a reliable and valid instrument that may be used to screen major depressive disorders among Afaan Oromo speaking Ethiopian pregnant women.

## Introduction

A recent literature review on prevalence and determinants of common perinatal mental health disorders by Fisher et al. revealed that only 8% of low and lower-middle-income countries (LMIC) have available data on the antenatal prevalence of common mental disorders, with most of this literature published after 2002 [1]. Furthermore, in nearly all studies, recruitment occurred during antenatal visits at a health facility, which precludes generalizability to women who do not have access to antenatal services. The limited evidence for LMIC, however, indicates that average prevalence is higher than in high-income countries (HIC)[1,2]. Fisher and her colleagues found that the prevalence of antenatal depression among women in LMIC is 15.6%, compared to 10% antenatal prevalence among women in HIC.

One of the reasons for the scarcity of perinatal mental health studies in LMIC may be the lack of validated scales for measuring depressive disorders in these countries. Most of the depressive disorders screening tools available today were developed for populations in HIC [3]. Previous studies showed that study participants in developing countries easily endorse somatic symptoms and are less willing to express emotional distress than people in developed countries [4,5]. In the case of pregnant women, this is further complicated by the similarity between selected symptoms of depression and the common experiences of pregnancy, including changes in sleep patterns, appetite, energy levels and concentration [6]. Furthermore, applying instruments developed in HIC to make conclusions about depression prevalence in LMIC may be inappropriate due to characteristics that are specific to LMIC, such as cultural differences and low literacy rates, which may affect the validity of depression screening tools [7–9].

In order to determine the adequacy of a measurement tool, one must assess the reliability and validity of the instrument. The reliability of the measurement tool can be determined by asking if the tool measures a variable in a consistent way. Validity can be assessed by determining if the instrument is an accurate measure of the underlying construct [10]. Flaherty et al. recommend five major dimensions of cross-cultural equivalence to be examined for validation of a psychiatric rating scale in a new cultural setting [11]. These dimensions include content, semantic, technical, criterion, and conceptual equivalence. After each type of validity is established in the first cultural setting, it must be reassessed in the second cultural setting. Few validation studies for scales assessing major depressive disorder (MDD) have been conducted in sub-Saharan Africa, and none in Afaan Oromo language[12,13]. In this paper, we present validation of the PHQ-9 for antenatal depressive symptoms screening in Oromo pregnant women in a primarily rural area of Ethiopia.

## Methods

### Setting

The study was conducted in Seka-Chekorsa District; a primarily rural area in Ethiopia situated 370 kilometers southwest of the capital Addis Ababa. In 2012, the District Health office reported a total population of 236,611, of whom 9062 (3.8%) were pregnant women. Afaan Oromo was spoken as a first language by 88.4% of the population in this District.

### Design

A descriptive cross-sectional study design was implemented to investigate the reliability and validity of the Patient Health Questionnaire (PHQ-9).

### Participants

Two hundred forty six respondents were recruited from six randomly selected *kebeles* (the smallest administrative unit in Ethiopia) in Seka-Chekorsa District. All the respondents agreed to take part in the study. The age of the participants ranged from 18 to 40 years (mean age 24.3 ± 5.6). All of the participants were married with average family size of 4.5 (range 2–11). The median gravidity and parity were 3 (range 1–10) and 2 (range 0–9), respectively. In each kebele, the participants were randomly selected from the Health Extension workers’ pregnant women registration book. This list of pregnant women is routinely updated by Health Extension workers in order to plan onsite and outreach services. The Health Extension workers directed selected pregnant women to their interview sites—commonly health posts, Kebele administration offices or school compounds.

The PHQ-9 questionnaire was administered to all 246 participants and 222 (90.2%) participants also volunteered to respond to the MINI-Plus (gold standard). Study participants who did not respond to the MINI-Plus did so simply because they needed to return home to attend routine responsibilities. In order to assess test retest reliability, one week after the first interview, the first 29 participating pregnant women completed the PHQ-9 questionnaire for a second time. The size of the retest sample (n = 29) was sufficient as suggested by Walter et al [14].

Eligibility criteria for participation included age 18 years and above, ability to communicate in Afaan Oromo language, informed consent, and lack of significant cognitive impairment that might interfere with the ability to participate in the interviews. Pregnant women less than 18 years old were excluded in order to avoid confounders due to medical and psychosocial issues unique to adolescent pregnancy.

### Instruments

#### Patient Health Questionnaire (PHQ-9)

The PHQ-9 is a 9-item self-administered questionnaire designed to evaluate the presence of depressive symptoms during the prior two weeks. The nine items of the PHQ-9 are based directly on the nine diagnostic criteria for major depressive disorder in the Diagnostic and Statistical Manual Fourth Edition (DSM-IV). The scale has the potential to serve as a dual-purpose instrument that may both screen for the presence of depressive disorder and assess the severity of symptoms [15].

Total PHQ-9 scores range from 0 (absence of depressive symptoms) to 27 (most severe depressive symptoms) to measure severity. Each of the nine items can be scored from 0 (not at all) to 3 (nearly every day). Major depression is diagnosed if five or more of the nine depressive symptoms have been present for at least “more than half the days” (a score of 2) during the past two weeks, and if one of the symptoms is depressed mood or lack of interest (anhedonia).

To date in sub-Saharan Africa, few studies have been published on the psychometric properties of the PHQ-9 [12, 16–18]. Only one study has been conducted in Ethiopia, and was conducted in Amharic language[12]. No PHQ-9 validation studies have been conducted in Afaan Oromo language.

#### MINI-International Neuropsychiatric Interview PLUS

The MINI-International Neuropsychiatric Interview (MINI-Plus) is a short structured diagnostic interview, developed jointly by psychiatrists and other clinicians, for diagnosis of the most common DSM-IV and ICD-10 psychiatric disorders [19].

For the purposes of this study, the English version of the MINI-Plus was translated into Afaan Oromo language by two native speaker mental health specialists. This version was later revised after involvement by a third specialist. The MINI-Plus includes 23 disorders, but for the current study only the modules for depressive disorders were used. The instrument was administered by two mental health specialists who were trained by a specialist with prior experience in applying the MINI-Plus instrument. Prior to the main study, the two raters interviewed 20 individuals using the MINI-Plus scale and agreed on the depression status of 18 respondents (90%). The inter-rater reliability showed substantial agreement (Kappa = 0.80, 95% CI: 0.519, 1.00, P<0.001).

## Semantic validation

Afaan Oromo is spoken by about 34% of the population in Ethiopia. Within Ethiopia, Oromo is the language with the largest number of native speakers [20]. The main dialects of Afaan Oromo in Ethiopia are Wellega (spoken in the West Wellega, East Wellega, Illubabor, and Jima zones), Tulama (in the North, West, and East Shoa zones), Wello (in Northern Shoa and Southern Amhara), Arsi (in the Arsi and Bale zones), Harar (in the West and East Harerge Zones), and Borena (in the southern-most zone by the same name). This validation study was conducted for Wellega and Tulama dialects.

### Translation

Translation and cultural adaptation of the PHQ-9 was performed according to ‘The Minimal Translation Criteria’ [21]. Two independent bilingual translators (psychologist and health education specialist) with advanced levels of English language and native Afaan Oromo language skills translated the questionnaire into Afaan Oromo (forward translation). With the contribution of a third reviewer with expertise in mental health, a reconciliation meeting was conducted to develop a consensus version (reconciliation Afaan Oromo version). An English language lecturer, who is a native Afaan Oromo speaker and who had been blinded to the original version, retranslated the reconciliated Afaan Oromo version into the source language (back translation). There were no major difficulties in reconciling the back-translated version.

### Cognitive debriefing

A cognitive debriefing process was applied for the cultural adaptation of the questionnaire as the last step of the translation procedure [21]. This process was carried out in order to identify any areas presenting linguistic problems and to assess the participants’ level of understanding in order to reveal inappropriate items and translation alternatives. As part of this process, the questionnaire was administered to 21 Afaan Oromo speaker pregnant women who were at different gestational ages. In the interview, all items were revised for comprehension (meaning and question objectives), information retrieval (type of information and recall strategy), decision process (sensitivity and social desirability), and adequacy of response options. Feedback was discussed in a debriefing summary before the final Afaan Oromo version of PHQ-9 was adapted.

The Afaan Oromo version of the PHQ-9 instrument was administered to 246 pregnant women by clinical nurses working in the psychiatry department of Jimma University specialized Hospital. Participants were then interviewed using the Afaan Oromo version of the MINI-Plus questionnaire by two mental health specialists who were blind to PHQ-9 results. In order to minimize order effects, respondents were randomized to receive the PHQ-9 or the MINI-Plus interview first.

## Statistical analysis

The data were analyzed using SPSS for Windows version 20 (Chicago, Illinois), Winsteps ver. 3.80.1 and STATA version 12. Descriptive characteristics were calculated for the socio-demographic variables. Reliability related to internal consistency was measured by Cronbach’s alpha coefficient (Cronbach’s α), while test-retest reliability was assessed by intra-class correlation coefficients (ICCs).

The sensitivity, specificity, accuracy, positive likelihood ratio and negative likelihood ratio were calculated for different cut-off scores of the PHQ-9 to construct a Receiver Operating Characteristic (ROC) curve. The area under the curve (AUC) was used to address the performance of a test. An AUC of 1.0 indicates perfect accuracy, while an AUC of 0.5 indicates a non-discriminating test. Youden Index, calculated as sensitivity plus specificity minus one and converted to a percent, was computed as an additional metric for cutoff determination. Although there are no empirical cutoffs for Youden Index, values above 50% are generally considered suitable values of diagnostic accuracy [22].

An exploratory factor analysis (EFA) was performed in order to determine the structure of the questionnaire [23,24]. The number of factors was determined with reference to the Kaiser criterion of Eigenvalues and the scree test [25]. A factor was considered important if its eigenvalues exceeded 1.0 [26].

Finally, Rasch analysis was conducted to substantiate the evidence suggesting the PHQ-9 scale is a reliable and valid tool for screening antenatal depression. The analysis was carried out according to the Andrich Rating Scale model [27] using Winsteps version 3.81.0 [28] to evaluate the operation of the response categories, to see how reliably respondents discriminated between response categories and to identify how well each item contributed to the underlying measure [29]. In Rasch analysis, the probability of an individual’s choosing a response on a particular item depends on both the person ability and item difficulty. For measurements assessing depression, “item difficulty” refers to the level of depression expressed by the item and “person ability” refers to the extent to which the study participants possess the depression [30,31].

### Ethical considerations

Ethical approval was obtained from the research ethical review board of Jimma University. Informed consent was obtained from each study participant and all interviews were conducted in private. Study participants who were screened as depressed or with suicidal attempts using the MINI-Plus were referred for psychiatric care.

## Results

The median PHQ-9 score was 4 (range 0–26) and items representing alterations in energy, sleep and appetite were the most commonly reported items, respectively. A total of 44 participants fulfilled DSM-IV criteria for MDD on the PHQ-9 (17.8%; 95% CI 13.0–22.6%). When interviewed by psychiatrists using the MINI-Plus questionnaire, a total of 28 participants (12.6%) fulfilled the DSM-IV criteria for MDD. PHQ-9 scores were higher among depressed individuals (mean = 13.5) compared to the non-depressed individuals (mean = 4.3).

### Reliability

Cronbach's alpha for the PHQ-9 total score was 0.84. The correlations between nine items of the PHQ-9 and the total scores ranged from 0.30 to 0.54, and all correlations were statistically significant (all 2-tailed p-values <0.01). One-week test-retest reliability of PHQ-9 total score was 0.98 for both agreement and consistency ICC indices. The mean PHQ-9 total score did not significantly increase over the two occasions (7.9 to 8.6, two-sided paired t test, p = 0.08). The quadratic and linear weighted kappa were 0.97 and 0.86 respectively for PHQ-9 severity categories (p < .0001).

### Validity

#### Semantic validity

This study’s translation of the PHQ-9 into Afaan Oromo favored the Wellega and Tulama Afaan Oromo dialects. Key words from the nine items of the questionnaire were first translated into Afaan Oromo words of many different dialects by the two translators. From these options, words commonly known in Wellega and Tuloma dialects were chosen during the consensus meeting. There were no major difficulties in reconciling the back-translated version.

#### Technical validity

To simplify administration of the PHQ-9 as an interview rather than a self-administered questionnaire, each statement was converted into question form. Besides, to provide reminders of the time interval for the recall, each item was introduced with a prefix ‘In the last two weeks…’. While participants understood the item scale and the two weeks recall period, they found it confusing to differentiate the response categories (e.g., “more than half the days” in reference to two weeks). To overcome this difficulty, a bar graph depicting the severity levels across response options was used in addition to reading the options. Respondents found the graph easier to understand than the verbal options.

#### Content validity

The cognitive debriefing revealed that the PHQ-9 was generally well understood, acceptable and culturally appropriate for all the respondents. However, interpreting opposite symptoms in items representing alterations in sleep, appetite and psychomotor agitation/retardation was challenging for the majority of participants who repeatedly asked how it would be possible to respond to two opposing events simultaneously. Upon hearing an explanation, participants understood that the items referred to changes in behaviors in either direction. To overcome this difficulty, these items were asked twice, each direction separately, and questionnaire administrators recorded the more severe response of the two.

#### Criterion validity

Table 1shows sensitivity, specificity, accuracy, positive likelihood ratio, negative likelihood ratio and Youden’s index for each of the PHQ-9 cut scores compared to the gold standard interview. As expected, sensitivity decreased progressively as the cut scores increased, with a marked decrease between the ≥8 and ≥9 cut scores (from 80.8% to 69.2%). In contrast, specificity between these two cut scores increased from 79.4% to 84.7%. Both Youden’s index and the cut scores of maximum sensitivity and specificity according to the ROC curve (Table 1) indicated the ≥8 cut scores as the most suitable for identifying pregnant women at increased risk of having depression. A total of 60 women (27.8%; 22.2–33.8%) scored ≥ 8 in the PHQ-9. Sensitivity at this cut score was 80.8% with specificity of 79.5%. The positive likelihood ratio at this point was 3.9 and negative likelihood ratio of 0.24.

**Table 1:** Detailed report of sensitivity and specificity of PHQ-9 among Afaan Oromo speaking Ethiopian pregnant women, 2017.

Cut point	Sensitivity	Specificity	Classified	LR+	LR-	Youden’s Index
(> = 5)	92.31%	57.37%	61.57%	2.1652	0.1341	49.68%
(> = 6)	88.46%	66.32%	68.98%	2.6262	0.1740	54.78%
(> = 7)	80.77%	74.74%	75.46%	3.1971	0.2573	55.51%
**(> = 8)**	**80.77%**	**79.47%**	**79.63%**	**3.9349**	**0.2420**	**60.24%**
(> = 9)	69.23%	84.74%	82.87%	4.5358	0.3631	53.97%
(> = 10)	65.38%	86.32%	83.80%	4.7781	0.4010	51.70%
(> = 11)	65.38%	89.47%	86.57%	6.2115	0.3869	54.85%
(> = 12)	61.54%	94.74%	90.74%	11.6923	0.4060	56.28%
(> = 13)	50.00%	94.74%	89.35%	9.5000	0.5278	44.74%
(> = 14)	46.15%	97.37%	91.20%	17.5384	0.5530	43.52%

**LR+ =** Likelihood Ratio Positive **LR- =** Likelihood Ratio Negative

**Fig 1:**
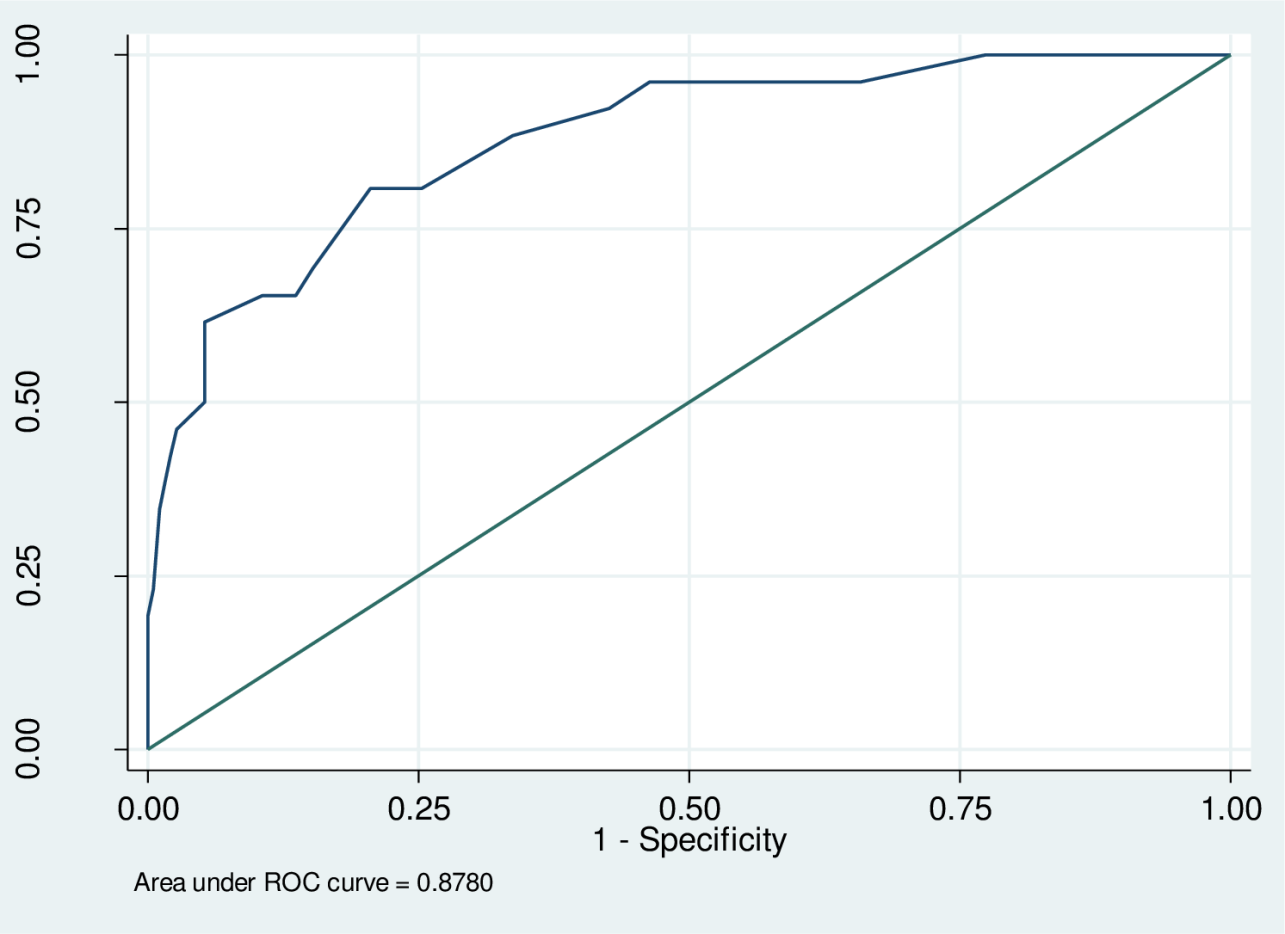
Receiver Operating Characteristic curves for PHQ-9 (n = 216).

The ROC curve, calculated for PHQ-9 is shown in Fig 1. The calculated AUC for the PHQ-9 score versus the MINI-Plus was 0.878 (SE = 0.036; CI = 0.807–0.949).

#### Construct validity (factor analysis)

The *Kaiser-Meyer-Olkin* (KMO) measure of the quality of the correlation matrix was high (KMO = 0.838). A significant Bartlett test of sphericity justified a dimension reducing procedure such as the factor analysis. The measure of sampling adequacy was greater than 0.80, so the items could be considered suitable for factor analyses. The scree plot revealed one dominant dimension with a big decrease between first and second eigenvalues and small decreases afterward (eigenvalues: 3.97, 1.02, 0.92, 0.71, 0.61, 0.53, 0.49, 0.45 and 0.30). Factor loadings ranged from 0.61 to 0.73 i.e., above 0.45 cutoff [32]. The percentage of total variance explained by the first factor was 44.1%.

### Rasch scale analysis

#### Rating scale utilization

The PHQ-9 meets the criteria established by Linacre [33] for rating scale effectiveness (Table 2). All category frequency counts are large (range from 189 to 1445) and the frequency difference is unimodal. All the average measures increase monotonically with rating scale category from -1.81 to -0.71 logits (a jump of 1.1 logits), from -0.71 to -0.23 logits (a jump of 0.51 logits), and then from -0.23 to 0.49 (a jump of 0.72 logits). Similarly, all categories have an acceptable mean-square (range from 0.78–1.12) indicating that the scale has a reasonably uniform level of randomness throughout the data. Furthermore, the step calibration -0.77, -0.04 and 0.80 are ordered and both the inference of measures-to-ratings and ratings to measures are generally strong and successful. Finally, step difficulties advanced by at least 1.4 logits (from -1.15 to +1.15 logits, a distance of 2.3 which is sufficiently large) and by less than 5.0 logits.

**Table 2:** Rasch analysis of the PHQ-9 scale.

Category label	Category		Average measure	Expected measure	Outfit calibration	Step calibration	Coherence M—> C	Coherence M—> C	Zone	
	Count	%							From	To
O	1445	65	-1.81	-1.76	0.96	-	84%	75%	-∞	-1.15
1	349	16	-0.71	-0.92	0.78	-0.77	31%	52%	-1.15	-0.02
2	231	10	-0.23	-0.20	1.12	-0.04	32%	34%	-0.02	1.15
3	189	9	0.49	0.60	1.11	0.80	71%	22%	1.15	+∞

#### Unidimentionality

Unidimentionality was assessed through analysis of Infit mean square (MNSQ) residuals, standardized Z (Zstd) values and point measure correlation and through principal component analysis.

**Fit Statistics:** Analysis of individual item fit revealed that all of the items were within the acceptable values for Infit MNSQ (0.93 to 1.28) and Zstd (less than 2.0). Moreover, the point measure correlation for the items (range from 0.44 to 0.72) indicated that the items were highly correlated with one another (Table 3).

**Table 3:** Item fit statistics of the PHQ-9 questionnaire using the Rasch analysis.

Entry number	Total score	Count	Measure	Model S.E.	Infit		Outfit		PTMEA corr.	Items
					MNSQ	Zstd	MNSQ	Zstd		
9	41	246	1.39	0.16	1.28	1.30	0.84	-0.40	A 0.44	PHQ9
4	294	246	-1.12	0.08	1.00	0	1.18	1.50	B 0.71	PHQ4
8	115	246	0.25	0.10	1.15	1.20	1.04	0.30	C 0.57	PHQ8
5	172	246	-0.26	0.09	1.07	0.70	1.12	0.80	D 0.62	PHQ5
1	151	246	-0.09	0.09	1.06	0.60	1.11	0.70	E 0.59	PHQ1
7	112	246	0.28	0.10	1.06	0.50	0.94	-0.30	d 0.58	PHQ7
6	105	246	0.35	0.11	1.05	0.40	0.76	-1.30	c 0.58	PHQ6
2	134	246	0.06	0.10	1.04	0.40	0.95	-0.30	b 0.60	PHQ2
3	254	246	-0.85	0.08	0.93	-0.70	0.90	-0.80	a 0.72	PHQ3

**Principal Component Analysis:** To judge the strength of the measurement dimension, the following cut off points were used for variance explained by the measure: >40% is considered a strong measurement dimension, >30% is considered a moderate measurement dimension, and >20% is considered a minimal dimension [34]. A ratio of 3 to 1 of variance explained by the measure to variance in the first contrast was also considered. As indicated in Table 4, variance explained by the measure is 47.2% which is strong principal measurement dimension. Secondly, 17.0% of the variance is explained by the first factor of residuals. The ratio of 47.2 to 17.0 is about 3 to 1 which is supportive of unidimentionality.

**Table 4:** Principal component analysis of standardized residual correlations for items (in eigenvalue units).

	Observed		Expected	
Total raw variance in observations	17.1	100.0%		100.0%
Raw variance explained by measures	8.1	47.2%		49.5%
Raw variance explained by persons	2.8	16.5%		17.3%
Raw Variance explained by items	5.2	30.7%		32.2%
Raw unexplained variance (total)	9.0	52.8%	100.0%	50.5%
Unexplained variance in 1st contrast	1.5	9.0%		17.0%
Unexplained variance in 2nd contrast	1.5	8.5%		16.2%
Unexplained variance in 3rd contrast	1.3	7.6%		14.4%
Unexplained variance in 4th contrast	1.2	6.8%		12.8%
Unexplained variance in 5th contrast	1.0	6.0%		11.5%

#### Item hierarchy

**Fig 2:**
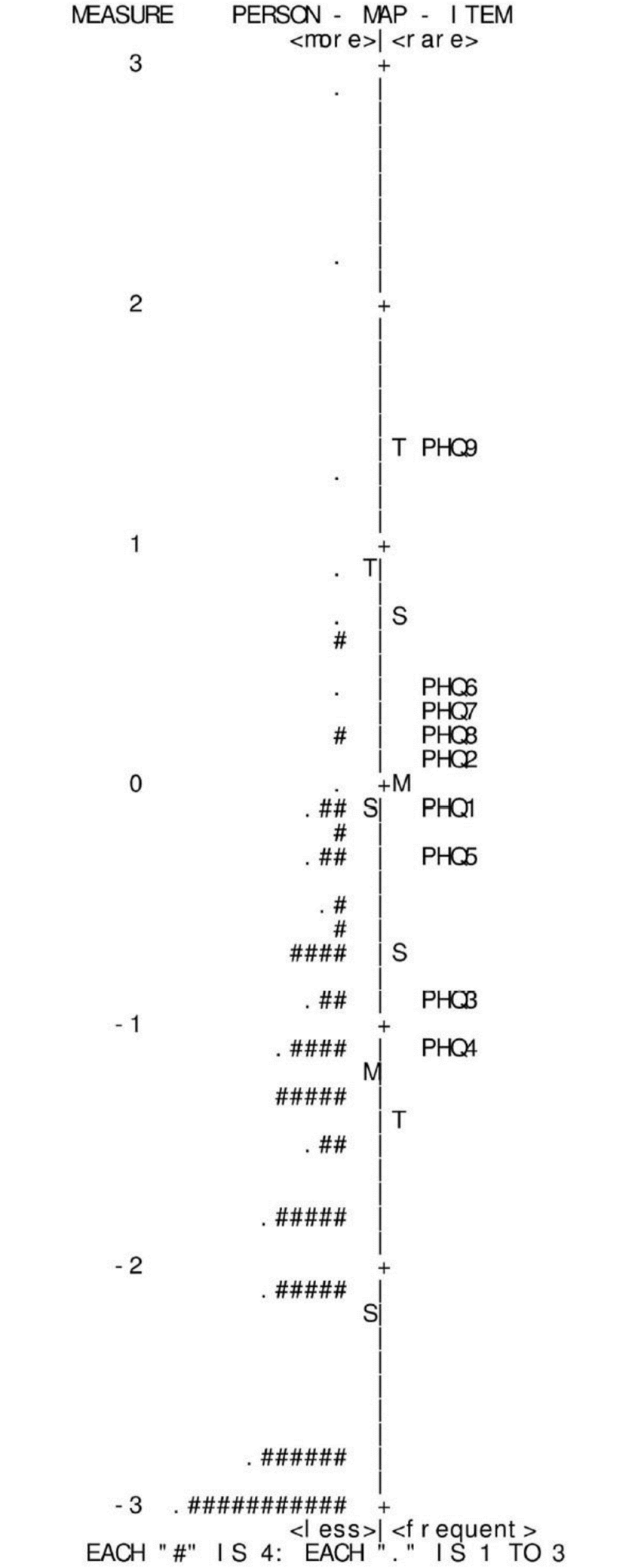
Person-item location map of the Rasch-scaled PHQ-9 showing the distribution of calibrated respondents’ scores (left hand side) and item locations (right-hand side).

Fig 2 represents the items in order of difficulty calibrated against person ability on a single interval scale, where the intervals are measured on the logit scale. The “Xs” on the left of the vertical axis corresponded to the person ability measures. As anticipated, there was an uneven spread of items across the full range of the participant’s scores, which indicates that most participants had low levels of depressive symptoms. The item hierarchy reveals that the item about suicide ideation was the most difficult to report whereas the items about trouble falling sleep and feeling tired were easier to report. The top factor (depressed mood, feeling of worthlessness, trouble concentrating and suicidal thoughts) is related to cognitive/affective symptoms. The bottom (trouble falling sleep, feeling tired or having little energy and appetite disturbance) concerns somatic symptoms [35]; these symptoms are common among pregnant women even when they are not depressed [6]. Thus, in general, somatic components of the items in the scale were easier to report than the affective components. However, item 8 (psychomotor agitation or retardation) which is a somatic symptom was difficult to report and item 1 (lack of interest) which is an affective symptom was comparably easier to report.

#### Floor/ceiling effects and item redundancy

In Fig 2, the Xs at the bottom left represent the 45 individuals (18.5% of sample) who rated all items 0 “not at all”. This exceeds the 15% cutoff point to state the presence of a floor effect [36]. It is likely that extreme items are missing in the lower end of the scale, indicating limited content validity. There was no ceiling effect (individuals who rated all item 3 ‘‘nearly every day”). No two or more items were situated at the same logit; thus, no redundant items were found in this study.

#### Internal consistency

Internal consistency was determined by examining separation index and reliability for persons and items (Table 5). In this study, the person separation index for the PHQ-9 is 1.54, indicating that the questionnaire categorized individuals into two distinct strata or levels of ability (depressed and non-depressed). Person reliability index, analogous to Cronbach’s alpha, was 0.70. The item separation index for the PHQ-9 is 6.54, which allows for categorization into 9 distinct strata. Item reliability index was 0.98. The high item reliability indicates that the relative order of item difficulty and the high reproducibility of the test items were consistent along the estimated continuum. Thus, the PHQ-9 demonstrates good internal consistency.

**Table 5:** Summary of 201 measured (non-extreme) person and 9 measured (non-extreme) item.

	Total score		Count		Measure		Model error		Infit				Outfit			
									MNSQ		Zsth		MNSQ		Zsth	
	P	I	P	I	P	I	P	I	P	I	P	I	P	I	P	I
MEAN	6.9	153.1	9	9	-1.15	-1.15	0.53	0.53	1	1	0.1	0.1	0.98	0.98	0.1	0.1
S.D.	5.3	73.6	0	0	1.03	1.03	0.2	0.2	0.54	0.54	1	1	0.8	0.8	0.9	0.9
MAX.	26	294	9	9	2.86	2.86	0.99	0.99	3	3	3.7	3.7	5.27	5.27	3.7	3.7
MIN.	1	41	9	9	-2.76	-2.76	0.35	0.35	0.15	0.15	-2.9	-2.9	0.18	0.18	-2.2	-2.2
REAL RMSE	0.6	0.11	TRUE SD		0.83	0.68	SEPARATION		1.38	6.19	PERSON RELIABILITY				0.66	0.97
MODEL RMSE			TRUE SD		0.86	0.68	SEPARATION		1.54	6.54	PERSON RELIABILTIY				0.7	0.98
S.E. OF PERSON MEAN = 0.07 (Person) S.E. OF PERSON MEAN = 0.24 (Item)																

ITEM RAW SCORE-TO-MEASURE CORRELATION = -.97 P = Person I = Item

## Discussion

To the authors’ best knowledge, this is the first validation of an Afaan Oromo version of the PHQ-9 questionnaire as a screening tool for depressive symptoms among pregnant women in Ethiopia. The main finding of this study is that the PHQ-9 scale has acceptable reliability and validity as a screening instrument for depressive symptoms among Afaan Oromo speaking Ethiopian pregnant women.

The strong internal consistency (Cronbach’s alpha = 0.84) suggests that the instrument is a highly reliable tool for screening depression in this study population. The acceptability of the internal consistency was further confirmed by the higher person and item separation reliability indices on the Rasch analysis. The test-retest reliability is higher than that of previous studies in sub-Saharan Africa [12, 17–19].

The cognitive debriefing revealed that the PHQ-9 was generally well understood, acceptable and culturally appropriate for all the respondents. However, interpretation of opposite symptoms in items 3 (trouble falling sleep), 5 (appetite disturbance) and 8 (psychomotor agitation or retardation) was challenging for the majority of participants. Williams et al. cited this obstacle as a potential limitation for the PHQ-9 in their 2009 study, noting that items containing polar opposite symptom descriptions may be difficult for some subjects to understand and could affect the psychometric properties of the PHQ-9 [37]. We suggest splitting these items to convert the PHQ-9 into PHQ-12 or asking these items forward, then backward, and scoring the more severe response as a symptom.

The mean scores on the PHQ-9 in the MINI-Plus depressed group versus the MINI-Plus non-depressed group were significantly different. This supports the construct validity of the PHQ-9. This screening tool also showed good criterion validity; the optimal cut-off value was eight. At this value, the PHQ-9 has a sensitivity of 80.8% and specificity of 79.5%. These values of sensitivity and specificity for the Afaan Oromo PHQ-9 are acceptable [38]. That means, 80.8% of pregnant women with depressive symptoms (according to the MINI-Plus), will be detected by the PHQ-9 and 79.5% of pregnant women without depressive symptoms by MINI-Plus will score negative on the PHQ-9. This finding is consistent with a meta-analysis [39] which reported that the PHQ-9 has acceptable diagnostic properties for detecting MDD for cut-off scores between eight and eleven. The pooled analysis revealed that specificity estimates summarized across 11 published studies ranged from 73% to 96% for PHQ-9 cut-off scores between 7 and 15.

An Amharic, hospital-based PHQ-9 validation study in Ethiopia showed that a threshold of ten was the most appropriate cutoff and offered the optimal discriminatory power in detecting MDD [12]. The relatively higher cutoff value reported in this study may be due to the medical patients over-reporting their somatic symptoms e.g. fatigue and anorexia, which could have resulted from their physical illnesses.

Despite few studies noting that PHQ-9 items may not accurately capture all components of MDD[30,37,40], the results of the Rasch analysis for this study did not detect item misfit using the mean infit and outfit square criteria set a priori. This is consistent with the Amharic PHQ-9 validation study in Ethiopia [12]. However, among the PHQ-9 items, item 3 (trouble falling sleep) and item 4 (feeling tired or having little energy) were easier to report while the question about suicidal thoughts (item 9) was the most difficult to report. This may be due to cultural values and reasons that impact reporting of certain depressive symptoms in this society [41]. Furthermore, suicidal thoughts indicate a more severe form of depression which is less common in community samples.

Both the factor analysis and the Rasch analysis revealed that a single factor model exists among the nine items of the PHQ-9 for Ethiopian pregnant women. This finding is consistent with previous studies that showed a single factor structure of the PHQ-9[12–14,30,39–44]. Thus, the PHQ-9 measures a single construct i.e. depressive disorder.

One of the limitations of the study is that participants may not represent all of the Afaan Oromo speaking population in Ethiopia because Afaan Oromo language has different dialects and the translation and cognitive debriefing were based on the Wellega and Tulama dialects. Additionally, as all of the assessments were conducted through interviews, technical validity-or comparison with self-administered formats-of the PHQ-9 was not assessed due to high illiteracy. Nevertheless, our findings support the utility of Afaan Oromo version of the PHQ-9 as screening tool for depressive symptoms during pregnancy in rural Ethiopia. The screening could potentially be integrated into routine home visits by rural Health Extension Workers.

In conclusion, the PHQ-9 has acceptable reliability and validity for screening of antenatal depressive symptoms and for measuring the severity of depressive symptoms for Afaan Oromo speaking rural Ethiopian pregnant women.
